# Knowledge, Perceptions, and Behaviors Regarding Antibiotic Use in a Community-Based Adult Sample in Salerno: An Observational Survey in a Province in Southern Italy

**DOI:** 10.3390/antibiotics14111081

**Published:** 2025-10-27

**Authors:** Emanuela Santoro, Raffaele Amelio, Roberta Manente, Giuseppina Speziga, Antonio Donato, Mario Capunzo, Giovanni Boccia

**Affiliations:** 1 Department of Medicine, Surgery and Dentistry “Scuola Medica Salernitana”, University of Salerno, 84081 Salerno, Italy; r.amelio@studenti.unisa.it (R.A.); gspeziga@unisa.it (G.S.); adonato@unisa.it (A.D.); mcapunzo@unisa.it (M.C.); gboccia@unisa.it (G.B.); 2 Clinical Pathology Unit, San Giovanni di Dio e Ruggi d’Aragona University Hospital, 84131 Salerno, Italy; manente392@gmail.com; 3 DAI Department of Health Hygiene and Evaluative Medicine, A.O.U. San Giovanni di Dio e Ruggi d’Aragona, 84131 Salerno, Italy; 4 U.O.C Hospital and Epidemiological Hygiene, San Giovanni di Dio e Ruggi d’Aragona University Hospital, 84131 Salerno, Italy

**Keywords:** antibiotic resistance, antibiotic knowledge, antibiotic perception, antibiotic stewardship

## Abstract

**Background/Objectives**: Antibiotic resistance represents one of the major global health emergencies, driven by the inappropriate use of antibiotics and persistent misconceptions among adults attending general medical clinics. This study, conducted on 325 participants recruited from general medical clinics in the province of Salerno, aimed to assess their knowledge, perceptions, and behaviors regarding antibiotic use. **Methods**: A cross-sectional, quantitative observational survey was conducted using a structured questionnaire based on the WHO tool and adapted to the local context. **Results**: The results show that the majority of participants take antibiotics only when prescribed by a doctor (90.2%), but risky practices such as self-medication (10%) and early discontinuation of therapy (16%) persist. In addition, 72% of subjects demonstrate incomplete knowledge about the independent management of drugs, and 86% mistakenly believe that resistance is limited to the individual rather than the community. The descriptive analysis stratified by age showed higher levels of awareness among subjects under 30 years of age, compared to significant knowledge gaps and inappropriate behaviors in the over-65 age group. **Conclusions**: Despite a good awareness of the need for medical prescriptions and the collective importance of the phenomenon, there are still critical areas of knowledge and incorrect practices that can promote the spread of antibiotic resistance. The data collected underscore the urgency of targeted educational strategies differentiated by age group, integrated with multi-channel communication interventions, in order to promote the appropriate use of antibiotics and contain the impact of one of the most serious global health emergencies.

## 1. Introduction

Antimicrobial resistance (AMR) is currently one of the most pressing challenges for global public health. The World Health Organization (WHO) estimated that, in 2019, bacterial AMR was directly responsible for 1.27 million deaths and contributed to an additional 4.95 million deaths worldwide [1]. In 2019, the six bacterial species most frequently responsible for deaths directly attributable to antibiotic resistance, in descending order, were Escherichia coli, Staphylococcus aureus, Klebsiella pneumoniae, Streptococcus pneumoniae, Acinetobacter baumannii, and Pseudomonas aeruginosa. Collectively, these pathogens accounted for nearly one million deaths, making infections caused by resistant bacteria the third leading cause of death worldwide, behind only ischemic heart disease and stroke [1]. To address this growing threat, in 2015 the World Health Organization (WHO) launched the Global Action Plan on Antimicrobial Resistance, endorsed by 193 countries, which calls for the establishment of international research programs, systematic progress monitoring, and the development of public awareness campaigns [2].

Given the severe impact of antibiotic resistance and the limited development of new antimicrobial agents, the World Health Organization (WHO) has established specific priority categories. Among the bacteria classified as critical priority are *Acinetobacter baumannii* and *Pseudomonas aeruginosa* resistant to carbapenems, as well as *Enterobacteriaceae* resistant to carbapenems and third-generation cephalosporins. Pathogens of high priority include *Enterococcus faecium* resistant to vancomycin, *Helicobacter pylori* resistant to clarithromycin, *Staphylococcus aureus* resistant to methicillin, *Shigella* spp. and *Campylobacter* spp. resistant to fluoroquinolones, *Haemophilus influenzae* resistant to ampicillin, and *Streptococcus pneumoniae* resistant to penicillin [3]. Recent evidence has highlighted that *Acinetobacter baumannii* remains among the most dangerous pathogens due to its high levels of resistance, which further increase clinical risk and complicate treatment [4]. Similarly, the so-called ESKAPE pathogens (*Enterococcus faecium*, *Staphylococcus aureus*, *Klebsiella pneumoniae*, *Acinetobacter baumannii*, *Pseudomonas aeruginosa*, and *Enterobacter* species) represent a growing threat in healthcare settings, as they are notorious for their ability to “escape” the action of most available antibiotics and cause infections that are difficult to treat [5]. Alongside the clinical aspects, the inappropriate use of antibiotics remains one of the main factors driving the spread of resistance, particularly in primary care settings and in the treatment of viral infections such as respiratory diseases [6]. Patients’ perceptions and expectations strongly influence prescribing decisions, while behaviors such as self-medication or the purchase of antibiotics without a prescription further exacerbate the problem [7]. Studies exploring public knowledge, attitudes, and behaviors regarding antibiotics and antimicrobial resistance (AMR) have revealed several consistent patterns, summarized as follows. Evidence indicates that, despite widespread awareness, major misconceptions persist most notably the belief in the efficacy of antibiotics against viral infections [8]. A systematic review conducted in Australia, the United Kingdom, and Sweden found that the public tends to underestimate its own responsibility in ensuring appropriate antibiotic use, and that public education campaigns, while necessary, are insufficient on their own to change consumption behaviors [9].

More recent qualitative research has shown that public perceptions of AMR are shaped not only by individual knowledge but also by ethical considerations, past experiences, doctor–patient dynamics, and the influence of the media and communication campaigns [10]. Public health campaigns therefore represent a potentially effective tool: a rapid review reported that well-designed initiatives, particularly those using mass media and targeted messaging, can raise awareness and promote more prudent antimicrobial use [6]. In Italy, recent studies have documented persistently high levels of antibiotic consumption among adults, along with the continued presence of inappropriate practices such as self-medication or premature discontinuation of therapy, often linked to incomplete or incorrect knowledge [7]. These findings highlight the urgent need to monitor and strengthen public awareness by promoting more effective and culturally tailored communication strategies, in line with the objectives of the WHO Global Action Plan [2].

In Italy, the WHO Global Action Plan has been translated into the National Action Plan on Antimicrobial Resistance (*Piano Nazionale di Contrasto all’Antimicrobico-Resistenza*, PNCAR 2017–2020), which was renewed for 2022–2025. Coordinated by the Ministry of Health, the PNCAR adopts a One Health approach and focuses on strengthening surveillance of antimicrobial resistance and antibiotic consumption, implementing antimicrobial stewardship programs in hospitals and primary care, enhancing the training and education of healthcare professionals, and promoting public awareness initiatives [11,12]. Despite these ongoing efforts, Italy continues to record some of the highest levels of antibiotic consumption in Europe, with marked regional disparities in the implementation of stewardship programs and awareness campaigns. These differences underscore the need for more consistent and effective nationwide application of the strategic framework [13].

## 2. Results

### 2.1. Sample Characteristics

**Figure 1:**
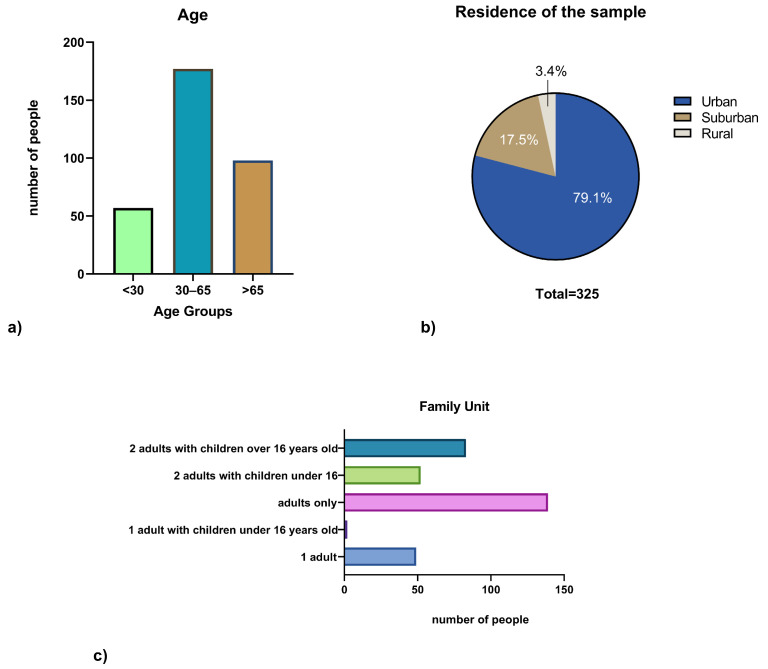
Sociodemographic characteristics of the sample. Panel (**a**) shows the distribution by age group; Panel (**b**) reports the area of residence, with a clear predominance of urban participants; Panel (**c**) displays the family unit composition.

The gender distribution was balanced, with 161 men (49.5%) and 164 women (50.5%) The sample was stratified into three age groups: younger than 30 years *(n* = 57; 17.5%), between 30 and 65 years (*n* = 170; 52.3%), and older than 65 years (*n* = 98; 30.2%). The distribution of the sample based on residence shows a prevalence of urban residents (*n* = 257; 79.1%), followed by suburban (*n* = 57; 17.5%) and rural (*n* = 11; 3.4%) participants. Family unit composition showed that the majority were adults living without children (*n* = 140; 43.1%), followed by two adults with children over 16 years (*n* = 80; 24.6%), two adults with children under 16 years (*n* = 50; 15.4%), individuals living alone (*n* = 50; 15.4%), and, to a lesser extent, single-parent families with children under 16 years (*n* = 5; 1.5%) (Figure 1).

### 2.2. Knowledge and Behavior Profiles

#### 2.2.1. Antibiotic Consumption

**Figure 2:**
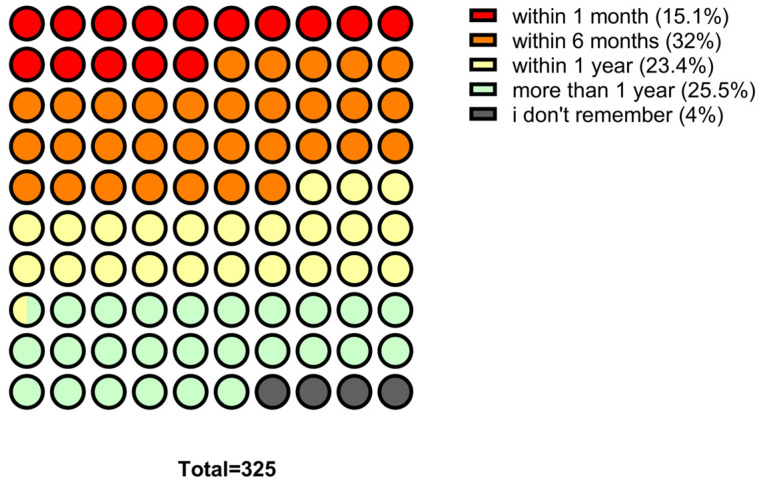
Distribution of the last antibiotic intake among participants.

**Figure 3:**
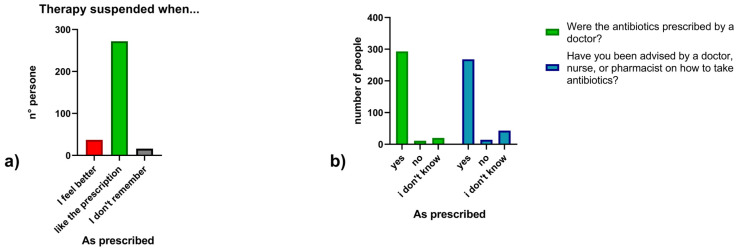
(**a**) Timing of antibiotic therapy suspension; (**b**) Medical prescription and counseling on antibiotic use.

**Figure 4:**
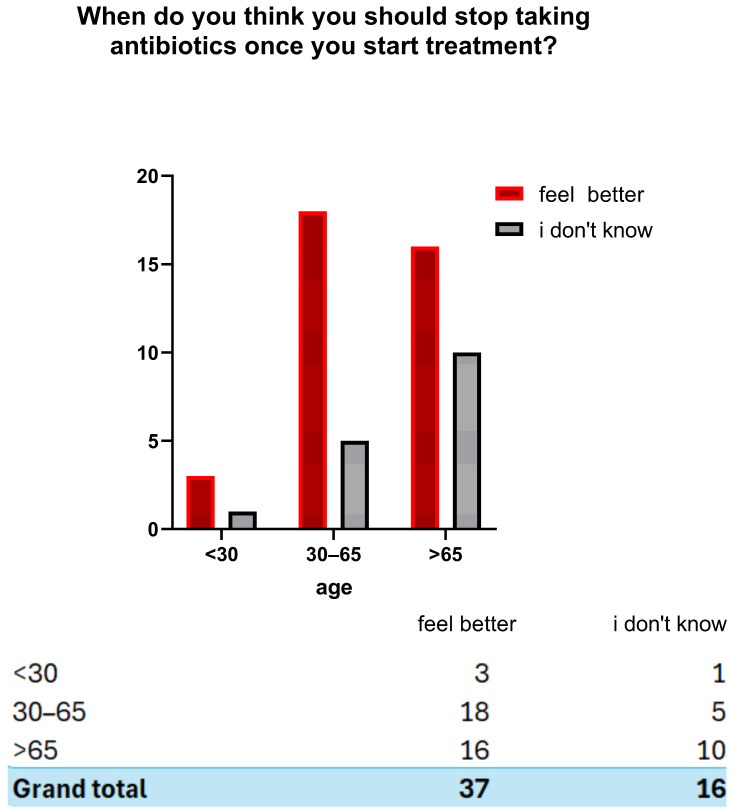
Perceptions of when to discontinue antibiotic therapy according to age group.

Regarding antibiotic use, 15.1% (*n* = 49) of participants reported taking antibiotics in the last month, 32.0% (*n* = 104) in the last six months, 23.4% (*n* = 76) in the last year and 25.5% (*n* = 83) more than a year ago, 4.0% (*n* = 13) could not recall. Overall, 70.5% (*n* = 229) reported at least one antibiotic course in the last year (Figure 2). Concerning prescriptions, 90.2% (*n* = 293) reported taking antibiotics on medical advice, 3.4% (*n* = 11) without a prescription, and 6.2% (*n* = 20) could not recall. In addition, 82.5% (*n* = 268) received instructions from a healthcare professional, while 4.3% (*n* = 14) did not receive instructions and 13.2% (*n* = 43) could not remember. (Figure 3) Regarding treatment duration, 83.7% (*n* = 272) answered correctly (completion of prescribed doses), 11.4% (*n* = 37) reported stopping when symptoms improved, and 4.9% (*n* = 16) did not know. The response “when I feel better” was more frequent among those >65 years 4.9% (*n* = 16), followed by those aged 30–65 years 5.5% (*n* = 18) and <30 years 3.1% (*n* = 3). Uncertainty was also higher among those >65 years (*n* = 10) (Figure 4).

#### 2.2.2. Knowledge About the Appropriate Use and Independent Management of Antibiotics

**Figure 5:**
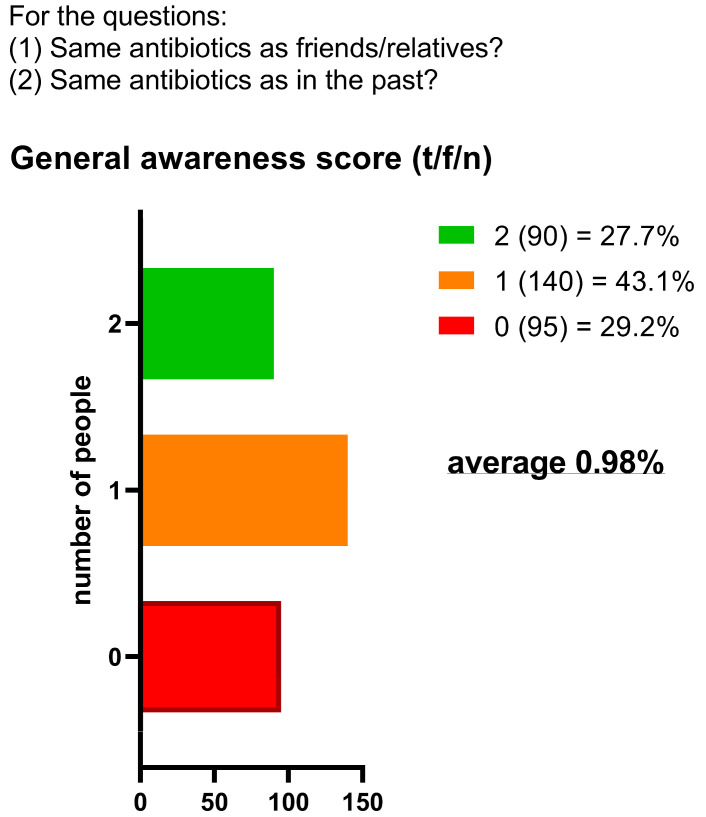
General awareness score about antibiotic use.

**Figure 6:**
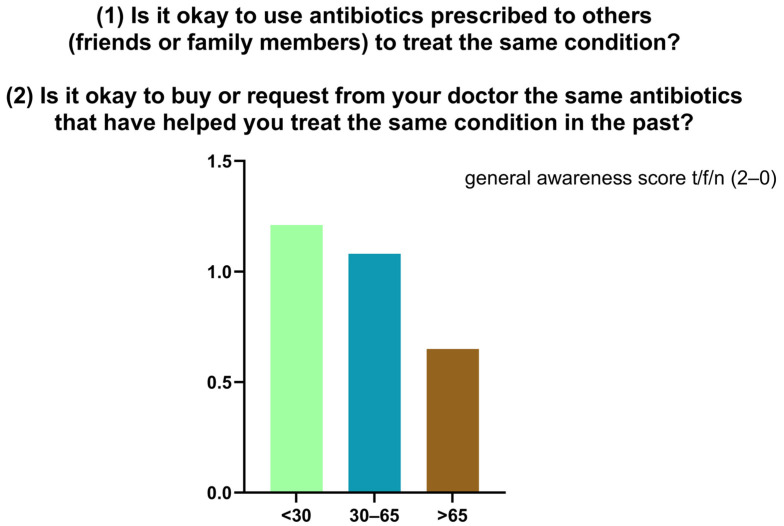
Distribution of general awareness scores regarding the reuse of antibiotics.

**Figure 7:**
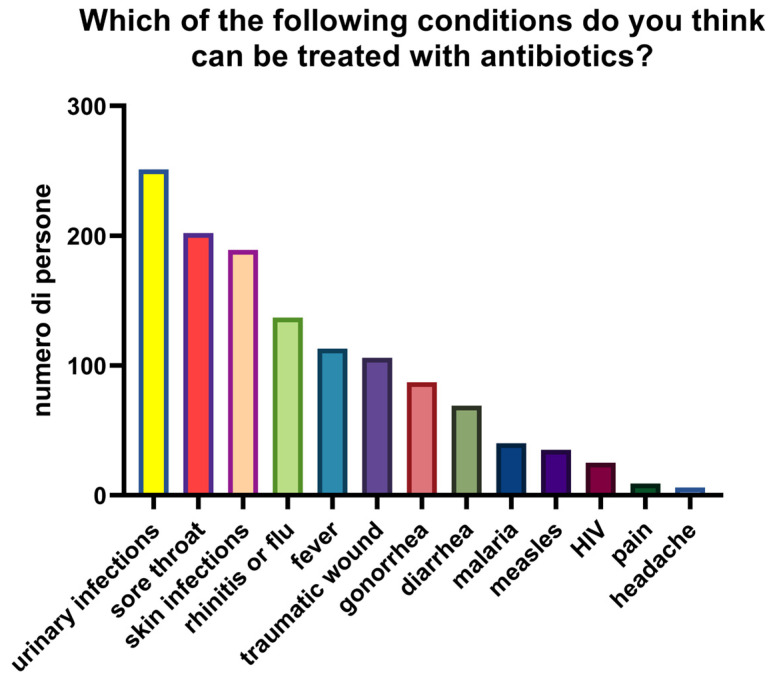
Conditions that participants considered treatable with antibiotics.

Knowledge of appropriate antibiotic use was limited. The mean score across two self-management questions was 0.98/2, with only 27.7% (*n* = 90) answering both correctly (Figure 5). To the first statement (“Is it okay to use antibiotics prescribed to others?”), 70.5% (*n* = 229) responded correctly (“false”) while 29.5% (*n* = 96) were incorrect or uncertain. To the second statement (“Is it okay to reuse antibiotics from past treatments?”), only 28% (*n* = 91) answered correctly, with 72% (*n* = 234) giving an incorrect or uncertain response. In the descriptive stratification by age, higher mean scores were observed among participants < 30 years (1.21), decreasing in the 30–65 years group (1.08) and in the >65 years group (0.65). (Figure 6). Participants reported perceived usefulness of antibiotics for urinary tract infections (*n* = 251), sore throat (*n* = 202), skin infections (*n* = 189), colds/flu (*n* = 137), and fever (*n* = 113). Misconceptions included use for traumatic wounds (*n* = 106), malaria (*n* = 40), pain (*n* = 9), and headache (*n* = 6) (Figure 7).

#### 2.2.3. Knowledge About Antibiotic Resistance

**Figure 8:**
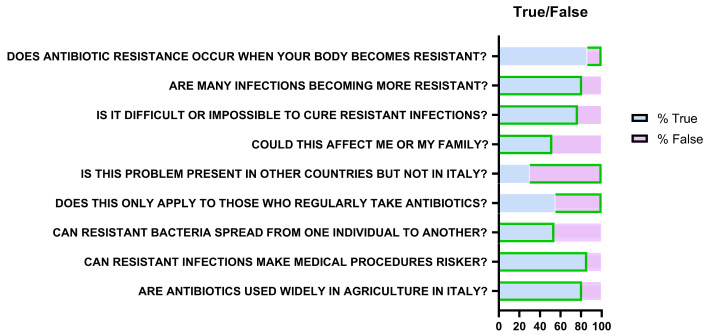
Representation of responses relating to knowledge about antibiotic resistance. Green borders indicate the correct answer (“True” or “False”) for each question.

True/false questions revealed mixed knowledge (Figure 8). Eighty-six percent incorrectly believed that resistance develops in the body rather than bacteria. However, 81% (*n* = 263) recognized increasing resistance of infections, 77% (*n* = 250) identified associated therapeutic difficulty, 52% (*n* = 169) acknowledged individual susceptibility, and 54% (*n* = 176) recognized transmission between people.

Seventy percent understood resistance is not limited to other countries but only 45% (*n* = 146) recognized it does not affect only frequent antibiotic users. Most participants acknowledged added risks for medical procedures (86%; *n* = 280) and antibiotic use in agriculture/livestock (81%; *n* = 263).

#### 2.2.4. Perceptions and Attitudes (Likert Scale)

**Figure 9:**
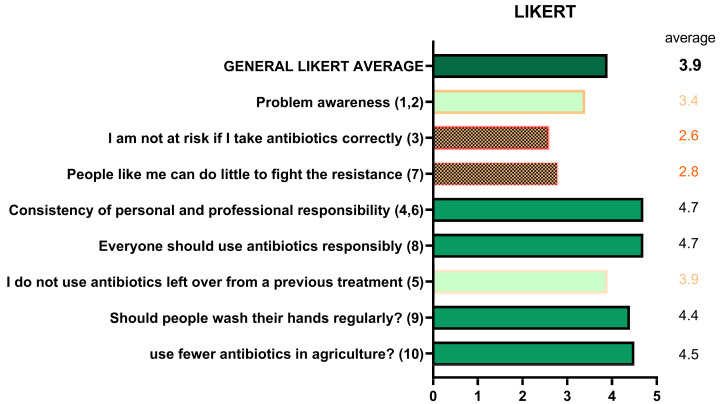
Perceptions and attitudes toward antibiotic use (Likert scale).

Responses to the ten Likert-scale items (1 = strongly disagree; 5 = strongly agree) provided insight into awareness, perceived risk, responsibility, and preventive behaviors (Figure 9). Awareness of the seriousness of AMR averaged 3.4/5, decreasing with age. The statement “I am not at risk if I take antibiotics correctly” scored 2.6, also decreasing with age. Personal responsibility scored low (2.8), while collective responsibility was widely acknowledged (4.7). Statements on prescriptions scored highly (4.7), as did those on advanced antibiotics (3.9). Preventive behaviors such as hand washing (4.4) and reduced antibiotic use in livestock (4.5) were also well recognized. Combined analysis of Likert and true/false results showed an inverse correlation with age: <30 years (Likert 4.04; true/false 6.21), 30–65 years (3.92; 5.89), >65 years (3.78; 4.68).

## 3. Discussion

Antibiotic resistance represents one of the greatest threats to global public health. The inappropriate use of antibiotics in both humans and animals promotes the selection of multidrug-resistant bacterial strains, leading to serious clinical and socioeconomic consequences [14]. According to recent estimates, resistant infections caused more than one million deaths in 2019, and projections suggest that this number could rise to 10 million deaths per year by 2050 if effective containment strategies are not implemented [15].

This study investigated knowledge, perceptions, and behaviors related to antibiotic use in a sample of 325 participants, providing valuable insights into the persistent critical issues observed within a heterogeneous population of primary care patients.

The majority of participants (over 90%) declared that they only take antibiotics on medical prescription, an encouraging figure in line with surveillance data previously conducted in Europe. In fact, according to data from Special Eurobarometer 522 on Antimicrobial Resistance (European Commission, 2022), almost all antibiotics used in Europe are still taken following a doctor’s prescription: 92% of respondents said they had received the drug from a healthcare professional, while only 8% reported using it without a prescription, often through drugs left over from previous treatments or obtained from acquaintances [16]. Nevertheless, approximately 10% of participants in our sample reported using antibiotics without consulting a doctor, indicating the persistence of risky self-medication practices. Particularly concerning is the proportion (16%) of individuals who discontinue antibiotic therapy when symptoms improve—a behavior known to favor the selection of tolerant bacterial strains, which can replicate, transmit resistance genes, and contribute to the overall spread of antimicrobial resistance [17]. These results are consistent with previous studies identifying poor treatment adherence as a key factor in the development of antimicrobial resistance. For instance, a survey conducted among university students in Israel revealed that a considerable proportion had discontinued antibiotic therapy before completion or had used leftover antibiotics from previous prescriptions—behaviors that directly contribute to the emergence of resistance. The authors emphasized that such practices are often linked to incomplete knowledge and misconceptions about how antibiotics function and how resistance develops, underscoring the importance of targeted educational interventions to address these misconceptions and promote better adherence to prescribed treatments [18]. One critical issue that emerged concerns the independent management of treatments: almost three-quarters of the sample demonstrated incomplete knowledge regarding the reuse of antibiotics already taken or prescribed to others. In particular, 37.5% believed it was correct to request the same antibiotic used in the past, confirming the persistence of misconceptions already documented in other populations. Such behaviors reflect the diffusion of a “self-prescribing culture” that calls for targeted educational interventions. Supporting this, a recent study in Saudi Arabia found that nearly 50% of respondents admitted to storing leftover antibiotics from previous prescriptions and reusing them later for symptoms like sore throat and fever [19]. Similarly, among Chinese parents, 48.1% stored antibiotics at home, with 63.1% of leftovers originating from earlier prescriptions—this was associated with a significantly higher likelihood (adjusted OR = 3.80, 95% CI: 2.89–5.00) of self-medicating their children [20]. Qualitative evidence from low- and middle-income countries further indicates that structural and cultural factors, including easy access to antibiotics and pervasive beliefs in their necessity, perpetuate this self-prescribing culture [21].

The questionnaire revealed substantial misconceptions: 86% of respondents incorrectly believed that antibiotic resistance is a property of the human body rather than of bacteria. This misunderstanding, previously reported in other studies, reduces the perception of collective risk and reinforces the notion that the problem is an individual one [22]. Conversely, participants demonstrated good awareness of the clinical severity of the issue—81% recognized the increasing incidence of resistant infections—and of the role of livestock farming and agriculture (81%), reflecting the growing adoption of the One Health perspective [23].

A recurring finding concerns age-related differences: descriptively, younger participants achieved higher scores on both objective knowledge questions and perception scales, whereas those over 65 exhibited more frequent knowledge gaps and less appropriate behaviors. These results are consistent with previous studies showing that older population groups are less exposed to recent awareness campaigns and more inclined toward self-medication practices [24]. Given their clinical vulnerability, this population should be considered a priority target for tailored educational interventions.

Overall, the data collected indicate that while theoretical knowledge regarding the proper use of antibiotics is widespread, it is often not translated into consistent behavior. Misconceptions persist (e.g., perceived effectiveness against viruses, reuse of leftover antibiotics), and personal risk tends to be underestimated. To address these gaps, it is essential to invest in clear and accessible communication campaigns, tailored to specific age groups, that explain in simple and concrete terms why the appropriate use of antibiotics represents an act of collective responsibility.

This study has several limitations that should be acknowledged. First, only descriptive statistical analyses were conducted; therefore, the findings are limited to observed trends and do not allow for the identification of statistically confirmed associations. Second, the cross-sectional design prevents causal inference and provides only a snapshot of the population at a specific point in time. Finally, the results are based on a sample from a single province, which may limit their generalizability to other contexts. Nevertheless, a key strength of this study is the use of a structured and standardized questionnaire adapted from the WHO tool, which ensures methodological rigor and enables comparability with international data.

## 4. Materials and Methods

### 4.1. Study Design and Participant Demographics

This study was designed as a quantitative cross-sectional observational survey conducted on a sample of 325 subjects recruited from general medical clinics in the province of Salerno. The tool used was a structured questionnaire developed on the basis of the WHO questionnaire, appropriately translated and modified in some parts to make it more usable at the local level. The inclusion criteria were age ≥ 18 years. For the purpose of analysis, subjects were categorized into three age groups: <30 years, 30–65 years, and >65 years. Participation was voluntary, subject to informed consent, and all questionnaires were completed anonymously.

### 4.2. Questionnaire Development and Structure

The survey tool was based on the questionnaire originally developed by the World Health Organization (WHO), “Antibiotic resistance: multi-country public awareness survey”. The questionnaire was adapted from the WHO tool ‘Antibiotic resistance: a multinational survey of public awareness’. The English version was translated into Italian and revised by the authors. Cultural adaptation involved minor modifications to wording and structure, aimed at improving clarity, contextual relevance, and alignment with the objectives of the study (e.g., replacing medical terms with commonly used equivalents).

The questionnaire administered, which is included as Supplementary Material, was divided into four main sections:

- Personal and socio-demographic section: collection of information on age, gender, place of residence and family composition;
- Behavioural section: exploration of personal practices regarding the use of antibiotics (last intake, self-prescription, methods of discontinuing therapy, reuse of previously prescribed antibiotics, intake of drugs prescribed to third parties);
- Knowledge section: assessment of basic knowledge on the appropriate use of antibiotics, the distinction between bacterial and viral infections, and the mechanisms of antibiotic resistance, using multiple-choice questions and “True/False” questions;
- Attitudinal and perceptual section: analysis of beliefs and perceptions regarding the use of antibiotics.

Responses of “I don’t remember” were reported as a separate category and included in the denominators for behavioral questions (e.g., prescription, discontinuation of therapy). For knowledge questions, “don’t know” responses were classified as incorrect in the calculation of knowledge scores.

### 4.3. Likert Scale and Scoring

Participants’ perceptions and attitudes towards antibiotic use were assessed using statements measured on a 5-point Likert scale, with values ranging from “strongly disagree” to “strongly agree”.

### 4.4. Data Collection and Analysis

The collected data were processed using descriptive statistical analysis. Qualitative variables were summarized using absolute frequencies and percentages. No inferential tests were performed, and results are presented as observed trends. Qualitative variables were summarized using absolute frequencies and percentages. The processing was carried out using Microsoft Excel (version 365, Microsoft Corporation, Redmond, WA, USA, 2024) and GraphPad Prism software (version 8.4.2; GraphPad Software, San Diego, CA, USA).

### 4.5. Ethical Considerations

Data collection and management were carried out in compliance with current legislation on personal data protection. All participants received comprehensive information on the objectives of the research and were given adequate time to read and understand the content of the questionnaire. The study was conducted in accordance with the ethical principles set out in the Declaration of Helsinki. Ethical approval was obtained from the Campania 2 Ethics Committee (protocol no. 2025/4722), which ensured that all procedures were carried out with respect to the safety, confidentiality and autonomy of the participants.

## 5. Conclusions

This study assessed knowledge, perceptions, and behaviors related to antibiotic resistance in a sample of adults attending general medical clinic. The results show a good awareness of fundamental principles, such as the importance of medical prescriptions and the recognition of collective responsibility. However, significant misconceptions persist, including the idea that it is the human body that develops resistance or that correct individual use is sufficient to protect against the phenomenon. There was also marked variability related to age: young people had higher levels of knowledge, while those over 65 were less informed, despite being more vulnerable to the clinical consequences of resistant infections. These results highlight the need for targeted and differentiated educational interventions for different age groups, combining accurate information with simple and contextual explanations. In particular, interventions should focus on:

(1) older adults, through tailored communication campaigns addressing misconceptions about resistance and therapy adherence;
(2) the involvement of general practitioners as key mediators in promoting appropriate antibiotic use;
(3) school-based educational programs to consolidate correct knowledge among younger populations.

Furthermore, multi-channel media campaigns should be developed to reach different age groups, pharmacists could be actively involved in patient education, and training initiatives for healthcare professionals should reinforce communication on the risks of self-medication and drug reuse.

Overall, the data confirm the urgent need for integrated communication and training strategies, with the active involvement of schools, the media, general practitioners, and pharmacies, in order to promote the responsible use of antibiotics and help contain one of the main threats to public health.

## Data Availability

The data presented in this study are available in this article.

## Supplementary Materials

The following supporting information can be downloaded at: https://www.mdpi.com/article/10.3390/antibiotics14111081/s1, File S1: Questionnaire template.
