# Supplementary material for: Knowledge, Perceptions, and Behaviors Regarding Antibiotic Use in a Community-Based Adult Sample in Salerno: An Observational Survey in a Province in Southern Italy
Source: Antibiotics (Basel). 2025 Oct 27;14(11):1081. doi: 10.3390/antibiotics14111081 (PMC12649112; doi:10.3390/antibiotics14111081)
Supplement: Supplementary file 1 [file antibiotics-14-01081-s001.zip › antibiotics-3891289-supplementary.pdf]

ANTIBIOTIC RESISTANCE QUESTIONNAIRE (date ....../....../...........)

AGE:

GENDER:

- ☐ **F**
- ☐ **M**

1) **How would you describe the place where you live?**
   - ☐ Urban
   - ☐ Suburban
   - ☐ Rural

2) **Which of these combinations best describes your family unit?**
   - ☐ 1 adult
   - ☐ 1 adult only and at least one child under the age of 16
   - ☐ Married/cohabiting - adults only
   - ☐ Married/cohabiting and at least 1 child under the age of 16
   - ☐ Only adults aged 16 or over
   - ☐ Several adults aged 16 or over and at least one child under the age of 16

3) **When was the last time you took antibiotics??**
   - ☐ In the last month
   - ☐ In the last 6 months
   - ☐ In the last year
   - ☐ More than a year ago
   - ☐ Never
   - ☐ I don't remember

4) **On that occasion, antibiotics were prescribed to you by a doctor?**
   - ☐ Yes
   - ☐ No
   - ☐ I don't remember

5) **On that occasion, you were advised by a doctor, nurse, or pharmacist on how to take antibiotics?**
   - ☐ Sì (per esempio: prima o dopo i pasti, per 7 giorni, ecc.)
   - ☐ No
   - ☐ I don't remember

6) **When do you think you should stop taking antibiotics once you have started treatment??**
   - ☐ When I feel better
   - ☐ When I took all the doses of antibiotics as prescribed
   - ☐ I don't know

7) **Is it okay to use antibiotics that have been prescribed to someone else (a friend or family member) to treat the same illness?**
   - ☐ True
   - ☐ False
   - ☐ I don't know

8) **If you have a specific illness, it is correct to buy or ask your doctor for the same antibiotics that have**

helped you combat the same symptoms in the past?

- ☐ True
- ☐ False
- ☐ I don't know

9) Which of the following diseases/disorders do you think can be treated with antibiotics? (More than one answer)

- ☐ HIV/AIDS
- ☐ Gonorrhea
- ☐ Bladder or urinary tract infections
- ☐ Diarrhea
- ☐ Colds and flu
- ☐ Fever
- ☐ Malaria
- ☐ Measles
- ☐ Skin infections
- ☐ Traumatic injury
- ☐ Sore throat
- ☐ Widespread pain
- ☐ Headache

10) Indicate which statements you think are true or false.

True	False	Antibiotic resistance occurs when your body becomes resistant to antibiotics and, as a result, antibiotics no longer have the desired effect.
True	False	Many infections are becoming increasingly resistant to antibiotics.
True	False	If bacteria are resistant to antibiotics, it can be very difficult, if not impossible, to treat infections caused by the same bacterial agents..
True	False	Antibiotic resistance is a problem that can affect me or my family.
True	False	Antibiotic resistance is a problem in other countries but not in Italy.
True	False	Antibiotic resistance is a problem that only affects people who regularly take antibiotics.
True	False	Antibiotic-resistant bacteria can spread from one person to another.
True	False	Antibiotic-resistant infections can make certain medical procedures, such as surgery, organ transplants, and cancer chemotherapy, more risky and dangerous.
True	False	In Italy, antibiotics are widely used in agriculture and in the breeding of animals intended for human consumption.

11) **Express your agreement/disagreement (from 1 to 5) with the following statements**

	I strongly agree.	Agree	Neither agree nor disagree	Disagree	Strongly disagree
Antibiotic resistance is one of the most widespread and serious problems in the world.	5	4	3	2	1
I am concerned about the effect that antibiotic resistance will have on my health and that of my family.	5	4	3	2	1
I am not at risk of antibiotic-resistant infections if I take antibiotics correctly.	5	4	3	2	1
People should only take antibiotics when prescribed by a doctor.	5	4	3	2	1
People should not use antibiotics left over from a previous treatment to treat other illnesses.	5	4	3	2	1
Doctors should only prescribe antibiotics when they are necessary.	5	4	3	2	1
People like me can do little to combat antibiotic resistance.	5	4	3	2	1
Everyone must use antibiotics responsibly.	5	4	3	2	1
People should wash their hands regularly.	5	4	3	2	1
Farmers should use fewer antibiotics in livestock farms for human consumption.	5	4	3	2	1

Original WHO questionnaire “Antibiotic resistance: multinational public awareness survey” translated into Italian and modified in some parts.
